# The true complexities of “standard” family practice visits unmasked: an observational cross-sectional study in Regina

**DOI:** 10.1093/fampra/cmae022

**Published:** 2024-04-19

**Authors:** Mackenzie M M Heidel, Adam T Clay, Megan Dash, Danielle Cutts

**Affiliations:** Department of Academic Family Medicine, College of Medicine, University of Saskatchewan, Regina, Canada; Department of Academic Family Medicine, College of Medicine, University of Saskatchewan, Regina, Canada; Department of Academic Family Medicine, College of Medicine, University of Saskatchewan, Regina, Canada; Department of Academic Family Medicine, College of Medicine, University of Saskatchewan, Regina, Canada

**Keywords:** access to care, compensation, electronic medical records, medical home/patient-centred medical home, primary care, quality of care

## Abstract

**Background:**

Many patients present to their family medicine clinic with more than one health concern, placing an increased demand on family physicians. Research into the average number of concerns per regular family medicine visit is limited. Recognition of the frequency that family physicians address more than one concern per visit and adapting practices accordingly is important for improving patient care.

**Objective:**

To examine whether family physicians routinely address multiple different patient concerns during a single visit and if this is influenced by patient demographics.

**Methods:**

This study was conducted at a multi-physician family medicine clinic in Regina, Saskatchewan, Canada. Five physicians contributed their 500 most recent charts, extending retrospectively from 1 June 2023, from in-person visits by patients over 18 years of age and billed as regular appointments without billed procedures. Each chart was reviewed for the number of concerns addressed in the visit.

**Results:**

Fifty percent of visits addressed more than 1 concern (range = 1–8). A generalized linear mixed model using Poisson distribution showed certain physicians (incident rate ratio [IRR]: 1.192, 95% CI: 1.087–1.307, *P* < 0.001) and adults older than 65 years compared to adults less than 40 years (IRR 1.151, 95% CI: 1.069–1.239, *P* < 0.001) were more likely to present with multiple concerns, but patient sex was not a significant predictor.

**Conclusions:**

Family physicians routinely address more than one concern per visit. Standard visit length and billing practices should be adapted to reflect this complexity.

**Key messages**

- Family physicians routinely address more than one concern per visit.
- Adults ≥ 65 years are more likely to present with ≥ 1 concern per visit.
- Visit length and billing should allow for multiple concerns per visit.

## Background

We have hypothesized that family physicians routinely address multiple different concerns for their patients during a single visit despite only being able to report on one to the “data pool” in Saskatchewan, Canada at the time of this research.^1–7^ While alternative payment models do exist in Canada they depend on location and whether physicians are contract or fee for service. Understanding the complexity of primary care visits helps to provide information about the quality of care provided to patients in the time allotted and informs both how we pay for physician services and how care teams are designed.

An extensive literature search of the Medline database revealed that minimal research has been done to determine the average number of concerns per regular family medicine visit. While limited, all the available research states that, on average, greater than 2 concerns are addressed per primary care visit.^1–7^ However, none of this available research addressed the complexity of family medicine in the context of the Canadian health care system. An American study by Beasley and colleagues found that physicians reported, on average, more problems than they charted (3.05 vs 2.82) and only billed for 1.97 problems per encounter on average.^2^ This not only points towards a trend in underreporting but also reflects the challenges physicians face when billing for more than one issue. In a 2001 analysis of American family practice visits, an average of 2.7 problems and 8 physician actions were observed per encounter.^4^ A trend towards underbilling for the number of issues addressed in each visit was also observed.^4^ Analysis of recorded general practitioner consultations in the United Kingdom revealed 2.1 concerns voiced per consultation.^6^ In adult primary care, the number of concerns addressed per visit has been found to have increased from 5.4 to 7.1 from 1997 to 2005 with visit duration also increasing during this period.^1^ In Norway, recorded general practice consultations found that 2.6 problems per visit were addressed on average with this number increasing to 3.3 with the exclusion of acute conditions.^3^ The analysis of 982 Texas family physician and patient encounters revealed a mean of 5.4 concerns addressed per visit, with a range from 1 to 16.^7^

An increased number of problems managed per visit was found to be associated with increased consultation length, despite many medical systems only allowing physicians to bill for one concern per visit regardless of the number of concerns a patient may have.^8^ Each additional concern addressed in a patient encounter with a family physician was found to increase the visit length by a mean of 2.5 minutes (*P* < 0.001).^4^ Luft and Liang dubbed the practice of addressing multiple issues per visit “Max-Packing” and found that it was “associated with 3.4% lower overall resource use, improved clinical quality metrics, and comparable patient experience (except for worse wait time ratings)”.^9^

We hypothesized that patient and physician demographics influence both how many concerns a patient presents with and how many concerns a physician can address in a single visit. An increased number of concerns per visit has been previously associated with older age and female sex.^3^ Older age and female sex are also associated with increased consultation rates and length.^8,10–14^ As increased consultation time has been associated with increased physician age, we hypothesized that patients seeing a family physician who has been practicing longer will present with more concerns per visit. As physicians develop more experience in clinical time management, they may rearrange their days to accommodate longer consultation times for those who need it.^13^

General practice patients value the ability to address all the health concerns they have in a single visit.^3^ As the Canadian health care system focuses on providing patient centred care, patient requests are increasing the demands on physicians.^15,16^ Recognition of the frequency that family physicians address more than one concern per visit, and adapting visit length and billing practices accordingly, is likely to result in fair pay for family physicians, decreased use of health care resources, and, most importantly, improved patient care.

## Methods

We analysed the clinical encounter notes from 2,500 general practice visits from 5 different family physicians at the same clinic, Victoria East Medical Clinic, in Regina, Saskatchewan, Canada. The study was approved by the Biomedical Research Ethics Board at the University of Saskatchewan (Bio-3978). All charts were accessed using Accuro EMR 2017.130, electronic medical record software.

There are 11 physicians in practice at the Clinic. The Clinic provides chronic disease management, pharmacist, dietician, community health nursing, radiology, psychology and counselling, referrals, sports medicine, an after-hours walk in clinic, and laboratory services. The Clinic provides care to approximately 20,000 patients and has been in service for over 30 years. This practice was identified by the senior author, and participating physicians were recruited by word of mouth to allow the use of their records for secondary purposes.

The family physicians involved in the study ranged in family practice experience from 5 to 25 years. Each physician contributed their 500 most recent charts from in-person visits that were by patients over 18 years of age, billed as regular appointments and did not have additional billing for procedures such as mole removal or ear syringing. Walk-in visits were excluded as they did not reflect longitudinal family medicine visits. Visits with no chart note logged were excluded. Three of the participating physicians were female, and 2 were male.

The chart review extended from 1 June 2023, retrospectively as far as was required to obtain 500 eligible charts for each physician, ending 8 November 2022. Each chart was analysed for the number of discrete concerns addressed in the visit. A concern was defined as “an issue requiring physician action in the form of a decision, diagnosis, treatment, or monitoring”.^3,4^ As well, “if separate problems merged into 1 at the end of a visit (e.g. fever and chest pain merging to pneumonia), then only 1 problem was to be listed”.^2^

All analysis was conducted with SPSS version 28 using a significance level of α = 0.05. We built several models to determine the effect of patient sex, age group (< 40 years, 40–64 years, and ≥ 65 years), and provider. Both generalized estimating equation and generalized mixed linear models can utilize count data using Poisson log-linear regression. Unlike generalized estimating equations, generalized linear mixed models explicitly model the within-subject correlation by using random effects. As a first step, we built a null generalized linear mixed model that contained the intercept and the grouping variable (physician) as a random effect, which allowed us to test the significance of the variance component for the grouping variable. The one-tailed significance value was 0.106, indicating that multilevel modelling was not needed to account for clustering by physician.^17^ As such, we created a generalized linear model containing patient age, patient sex, and physician as fixed effects. As this model did not include random effects, the calculated incident rate ratios are equivalent to those that would be generated using generalized estimating equations. Finally, a separate generalized linear model was created using to test for interactions between gender and age group. This final model is not shown as the interaction terms were non-significant, and the model with interactions did not fit as well as the one without interactions (higher Alkaike’s Information Criterion).

## Results

The retrospective review of 2,500 visits resulted in 1,746 unique participants. Demographic information for patient visits is provided in Table 1. Fifty percent of the visits addressed more than one complaint (Table 1). The number of concerns addressed per visit ranged from 1 to 8, with a mean of 1.8 concerns per visit. Patient’s age and physician were significant predictors of the number of complaints raised during a visit (Table 2). Sex was not a significant predictor of multiple concerns.

**Table 1.** Characteristics of 2,500 Canadian in-person family medicine visits billed as regular appointments (2023).

		Frequency	%
Sex	Male	797	31.9
	Female	1,703	68.1
Age (years)	<25	140	5.6
	25–44	773	30.9
	45–64	890	35.6
	65–84	635	25.4
	>84	62	2.5
Number of concerns per visit	1	1,244	49.8
	2	752	30.1
	3	358	14.3
	4	117	4.7
	5	20	0.8
	6	5	0.2
	7	3	0.1
	8	1	0.0

**Table 2.** Impact of patient age, sex, and provider on having more than one concern per in person family medicine visit billed as a regular appointment including 2,500 Canadian visits (2023).

Model term	Incidence rate ratio	95% CI	*P*-value
Age			
< 40^a^	1	-	-
40-64 years	1.037	0.961–1.120	0.350
≥ 65 years	1.151	1.069–1.239	< 0.001
Provider			
Provider 1^a^	1	-	-
Provider 2	0.981	0.892–1.078	0.686
Provider 3	1.192	1.087–1.307	< 0.001
Provider 4	0.929	0.840–1.027	0.152
Provider 5	1.043	0.950–1.146	0.376
Male sex	0.997	0.931–1.068	0.936
Intercept	1.618	1.489–1.758	< 0.001

^a^Reference category.

## Discussion

Our study is the first of its kind to address the complexity of community family medicine in the Canadian context with respect to the number of concerns addressed per visit. Our results are consistent with previous research as we found that the majority of family medicine visits address more than one concern.^1–7^ Female sex was not found to be associated with multiple concerns per visit, consistent with some previous research.^2,5^ However, Bjørland and Brekke observed a relationship between female sex and presenting multiple concerns per visit.^3^

The association between increasing age and multiple concerns per visit is supported by earlier studies.^2,3,18^ Beasley et al. found visits with patients over 65 years of age address 3.88 concerns, as compared to a whole sample mean of 3.05.^2^ When examining elderly primary care visits, Tai-Seale et al. found that physicians addressed a median of six topics.^18^

Our study found that Provider 3, who was a male physician with the least practice experience at 5 years, addressed the most issues per visit. This differed from our prediction that patients seeing family physicians who has been practicing longer would present with more concerns per visit as the physician has more experience in clinical time management and might be able to rearrange their day to accommodate a longer consultation time for those who need it. Fewer years in practice could be associated with increased concerns addressed per visit due to the provider being less experienced with setting time boundaries with their patients. As well, as physicians gain practice experience, it is possible that their charting detail decreases resulting in less concerns charted per visit.

The current Saskatchewan family medicine billing system is constructed on the incorrect notion that each visit addresses only one issue.^19^ For example, if a patient comes in in need of one prescription renewal, this is billed for the same amount as if a patient comes in needing a prescription renewal, diagnosis of a cough, and musculoskeletal pain. The provincial medical systems need to adapt their billing technologies. Increasing appointment length to accommodate multiple concerns per visit as patients value the ability of physicians to address all their health concerns in a single visit would be helpful.^3^ The structure of physician compensation should be adapted to reflect the complexity of the visit. These changes are especially important as our population ages and more people have complex health concerns with multiple concerns to address in their family medicine visits. Alternative payment models have already been implemented in Canada. For example, the British Columbia Longitudinal Family Physician Payment Model compensates family physicians for “time, patient interactions, and the number and complexity of patients in their practice” as of 1 Februrary 2023.^20^ This model includes time codes for direct patient care, indirect patient care, and clinical administration.^21^ Alternatively, Ontario offers capitation-based payment models, which pay physicians per patient to deliver primary care services.^22^ Nova Scotia piloted a blended capitation model starting in 2022, which compensated family physicians based on the number of patients, number of services, and timely access to care.^23^ It is important to note that alternative payment models are associated with increased recruitment and retainment of family physicians,^24^ of which we have a national shortage.^25^

The ability to accommodate more than one concern per standard family medicine visit could be realized through the widespread implementation of team-based care structures such as the Patient’s Medical Home vision.^26^ This vision prioritizes interdisciplinary collaboration among health care professionals and has been associated with higher quality and more timely family medicine visits.^26^ As our health care system evolves to further prioritize patient-centred care, the Patient’s Medical Home vision could be a valuable tool, in conjunction with changes in billing practices and compensation strategies, to help physicians better address more the one complaint per visit.

### Strengths and limitations

The main strength of our study is the statistical power associated with the retrospective analysis of 2,500 patient charts. This retrospective chart analysis allowed us to analyse the impact of both provider and patient characteristics on family medicine visit complexity. Conversely, our study is limited by the accuracy and thoroughness of physician charting. If the physician was unable to completely chart all issues addressed in the visit, this would result in underreporting within the Accuro patient data. A trend towards underreporting among primary care patient charts has been well researched and could account for a falsely low percentage of family medicine visits addressing multiple concerns.^2,4^ Physician actions could encourage patients to bring up less concerns per visit. As well, this study only explored patients from 5 physicians at a single clinic in Regina, Saskatchewan, which limits the generalizability of results. Future iterations of this research should consider using multiple trained researchers to analyse all charts to ensure concern count accuracy and increase the sample size of physicians. As well, as Artificial Intelligence improves and becomes more integrated within the electronic medical records for charting functionality, future research should explore its use in mitigating charting bias and underreporting.

## Conclusion

Most standard family medicine visits address more than one concern. However, the Saskatchewan medical billing system only allows physicians to report one concern per visit to the data pool and standard visit lengths are recommended on the premise of addressing a single concern.^19^ Patients value the ability to have all their health concerns addressed in a single visit, and this structure is associated with lower use of health care resources and improved clinical quality.^3,9^ Our study is the first step toward recognizing the frequency with which Canadian family physicians address more than one concern per visit and supports the future adaptation of visit length, billing practices, and physician compensation structure to reflect this complexity. Future research should consider an increased sample size of physicians to further examine the impact of physician characteristics on the number of patient concerns per visit. As our health care system evolves to further prioritize patient-centred care, the Patient’s Medical Home vision could be a valuable tool, in conjunction with changes in billing practices, to help physicians better address more than one complaint per visit.

## Data Availability

The data underlying this article cannot be share publicly due to privacy concerns as the data is extracted from individual’s confidential medical record. The data will be shared on reasonable request to the corresponding author.
